# Inflammatory Myofibroblastic Tumors in Paranasal Sinus and Nasopharynx: A Clinical Retrospective Study of 13 Cases

**DOI:** 10.1155/2018/7928241

**Published:** 2018-10-15

**Authors:** Zhenzhen Zhu, Yang Zha, Weiqing Wang, Xiaowei Wang, Yali Gao, Wei Lv

**Affiliations:** Department of Otolaryngology-Head and Neck Surgery, Peking Union Medical College Hospital, Chinese Academy of Medical Sciences, Peking Union Medical College, No. 1, Shuaifuyuan, Wangfujing, Dongcheng District, Beijing 100730, China

## Abstract

**Background:**

Inflammatory myofibroblastic tumor (IMT), as a mesenchymal tumor, is common in the lung and abdomen but rare in the paranasal sinus and nasopharynx.

**Objective:**

This study aimed to summarize the clinical characteristics of IMT in the paranasal sinus and nasopharynx and analyze the relationship between the treatment and the overall survival (OS).

**Method:**

The clinical features, treatment, and follow-up data of patients diagnosed with IMT of the paranasal sinus or nasopharynx from 2006 to 2017 were retrospectively analyzed, and the previous literature was reviewed.

**Results:**

IMT often presents as an ill-defined soft-tissue mass with bone destruction and invasion of surrounding structures. The treatment methods used in this study were different combinations of surgery, prednisone, radiotherapy, and chemotherapy or observation alone. Three of the 13 patients were lost and the follow-up time of the remaining 10 cases ranged from 2 to 87 months (median, 39 months). Two patients died of the disease; the other eight patients were stable. The 5-year survival rate was 72%. Among the four methods of treatment, only treatment with prednisone was significantly correlated with better OS (*P* = 0.046).

**Conclusions:**

IMT is an intermediate tumor that often mimics malignancy. We are not sure if IMTs in the nasal cavity are more aggressive because of the biology or if the location and local therapy in the head region is more complicated. Radiologic findings help know the extent of the lesion. For unresectable nasal IMT, combined therapy with glucocorticoids, chemotherapy, and radiotherapy is sometimes a better choice. Glucocorticoids are especially recommended as a basic part of the integrated therapy. However, the standard treatment needs further research.

## 1. Introduction

Inflammatory myofibroblastic tumor (IMT) is recognized as an intermediate mesenchymal tumor by the World Health Organization [1]. Previously, IMT was also described as inflammatory pseudotumor, plasma cell granuloma, and inflammatory myofibrohistiocytic proliferation. It is characterized by myofibroblastic and fibroblastic spindle cells and an infiltrate of plasma cells, lymphocytes, and/eosinophils. Whether IMT is reactive or neoplastic in nature has been controversial, but recently it is considered as a true tumor because of the identification of anaplastic lymphoma kinase (ALK) gene rearrangement [2]. The etiology of IMT is uncertain, including ALK gene rearrangement, virus infection, trauma, and chronic inflammation [3]. IMT can be found anywhere in the body, with the lung being the most common organ involved. The proportion of head and neck involved in extrapulmonary IMT ranges from 14% to 18% [4]. IMTs of the paranasal sinus and nasopharynx are rare, and most studies were case reports. Meanwhile, no unified and standardized treatment for IMT is available to date. This study aimed to summarize the clinical characteristics of 13 patients with IMT in the paranasal sinus or nasopharynx and analyze the relationship between the treatment and the prognosis.

## 2. Methods

The medical records of 13 patients diagnosed pathologically with IMT, inflammatory pseudotumor, or plasma cell granuloma of the paranasal sinus and nasopharynx in the Department of Otolaryngology–Head and Neck Surgery, Peking Union Medical College Hospital from 2006 to 2017 were reviewed. All the specimens were diagnosed by experienced pathologists. The medical history, radiologic images, histopathological diagnosis, immunohistochemical results, treatment, and follow-up data were summarized. SPSS 23.0 was used for the statistical analysis. The Kaplan-Meier method was used for the survival analysis. A* P *value < 0.05 was considered significant. This study was permitted by the Ethics Committee of Peking Union Medical College Hospital.

## 3. Results

### 3.1. Clinical Characteristics

The records of 13 patients with IMT of paranasal sinus or nasopharynx diagnosed in the hospital were reviewed. The clinical characteristics of the patients were presented in Table 1. There was no gender difference in our cases, with six male patients and seven female patients. All of them were adults, with the age ranging from 21 to 64 years (mean 40.7 ±13.5, median 37). The symptoms were varied as determined by the specific location of the lesion. Among the 13 patients, the most common symptom was facial swelling and pain (*n* = 9). Other local symptoms included trismus (*n* = 6), headache (*n* = 5), toothache (*n* = 5), decreased vision (*n* = 4), diplopia (*n* = 3), and proptosis (*n* = 3). Besides the local specific symptoms, some common systemic symptoms were also observed, such as fever (*n* = 3), weight loss (*n* = 6), and anemia (*n* = 5).

### 3.2. Radiologic Findings and Abnormal Laboratory Results

**Figure 1:**
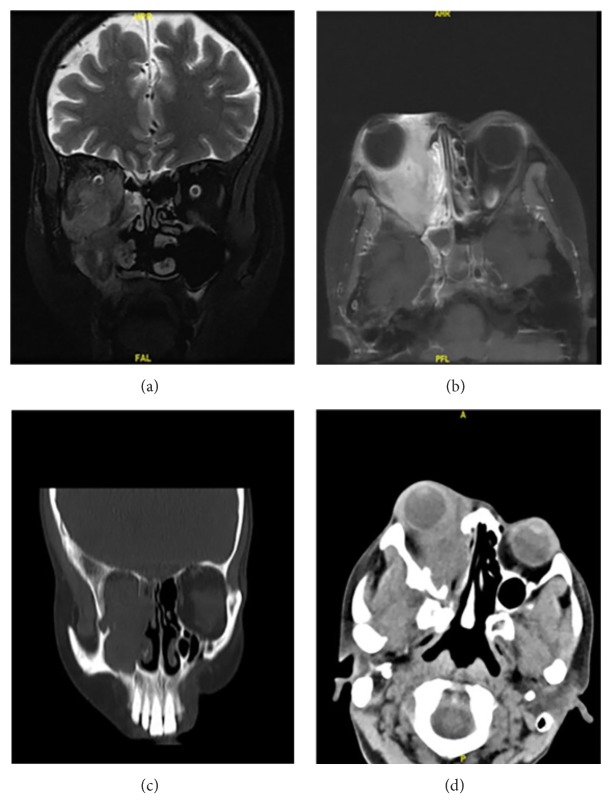
A 33-year-old woman complained of facial swelling, diplopia, and impaired vision. CT and MRI showed a soft-tissue tumor in the right maxillary sinus and orbit. Six years ago, she had been diagnosed with plasma granuloma of the right middle ear. This time she was diagnosed with an inflammatory myofibroblastic tumor and underwent endoscopy-guided incomplete resection, combined with prednisone and chemotherapy. After 1-year follow-up, her symptoms were significantly relieved. T2-weighted (a) and CT (c) coronal images showed a hyperintense and isodense mass in the maxillary sinus and orbit. Axial contrast-enhanced T1-weighted image (b) and CT (d) showed the intense enhancement of the soft-tissue tumor.

**Figure 2:**
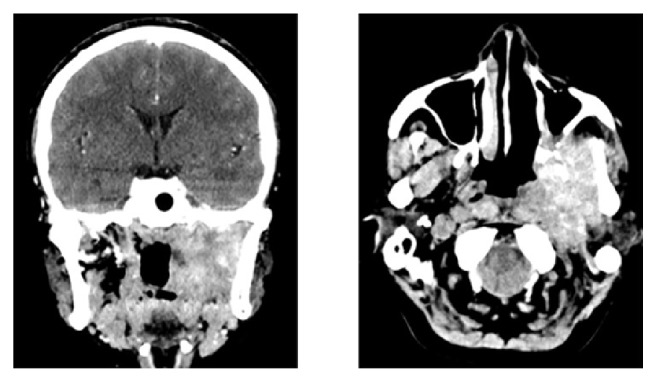
A 27-year-old woman complaining of head pain and trismus was diagnosed with IMT of the parapharyngeal space. Several biopsies were conducted before the diagnosis was confirmed. The contrast-enhanced CT showed the intense enhancement of the tumor surrounding the important vessels. She did not opt for the treatment after being informed of the benefits and risks of different treatments. After 37-month follow-up, no symptomatic progression was seen.

**Figure 3:**
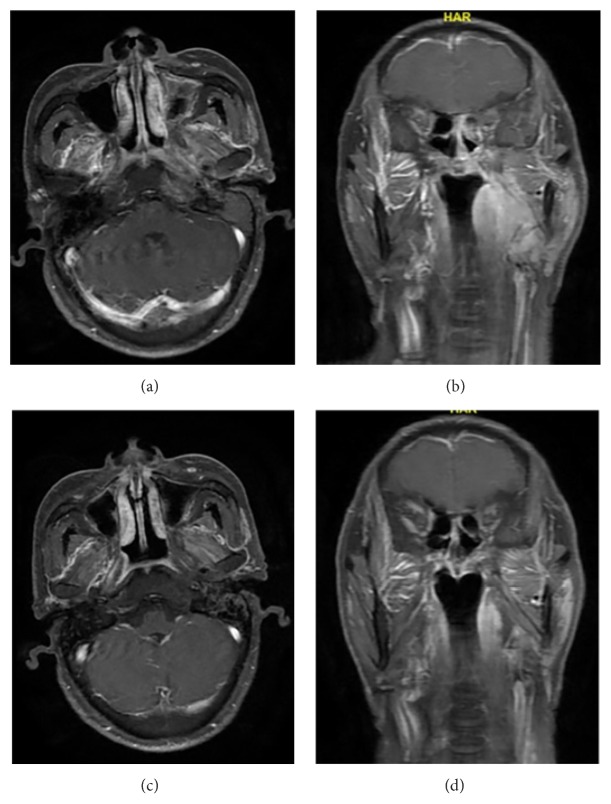
A 57-year-old man diagnosed with nasopharyngeal IMT received radiotherapy and oral steroids. After 22-month follow-up, both clinical and imaging manifestations improved. The images before treatment were shown above ((a) and (b)) and that 16 months after radiotherapy were shown below ((c) and (d)) in the figure.

**Figure 4:**
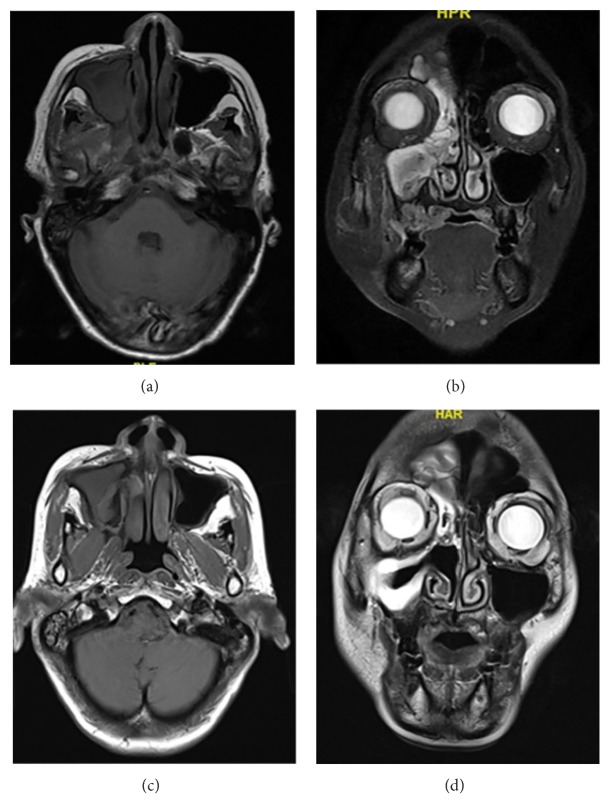
A 64-year-old woman was diagnosed with IMT of the nasopharynx and paranasal sinus. She underwent incomplete resection of tumor, combined with oral prednisone and radiotherapy. After 20-month follow-up, both clinical and imaging manifestations improved. The axial and coronal MRI images before treatment were shown above ((a) and (b)) and that 9 months after radiotherapy were shown below ((c) and (d)) in the figure.

**Figure 5:**
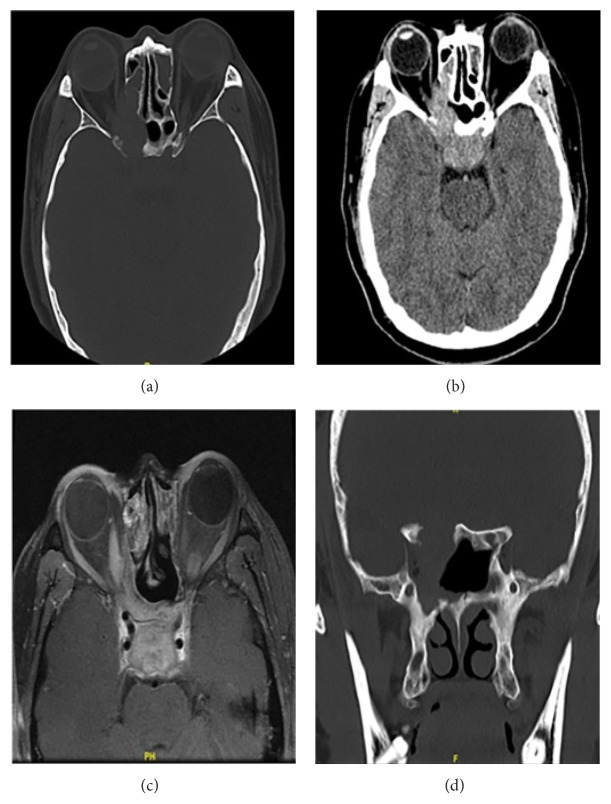
A 37-year-old man complained of decreased vision. Further imaging found an extensive soft-tissue tumor involving the sinus, orbit, and skull base with bone destruction. He was diagnosed with IMT after biopsy. However, he was lost to follow-up unfortunately.

The typical computed tomography (CT) and magnetic resonance imaging (MRI) images were shown in Figures 1–5. Invasive growth of the soft-tissue tumor with bone destruction and mild-to-intense enhancement in contrast-enhanced CT or MRI was observed. After treatment, an improvement was noted not only in symptoms but also in radiologic imaging in some patients (Figures 3 and 4). After reading the images, it was inferred that the most common site in the patients was maxillary sinus found in 10 patients. Other common sites included orbit (*n* = 9), ethmoid sinus (*n* = 8), nasal cavity (*n* = 5), infratemporal fossa (*n* = 5), pterygopalatine fossa (*n* = 4), parapharyngeal space (*n* = 4), sphenoid sinus (*n* = 4), and nasopharynx (*n* = 4) (shown in Table 1). Subcutaneous and intracranial involvement occurred in a few cases. The enlargement of head and neck lymph nodes was found in six patients. Only one was proved to be with IMT of lymph node pathologically, whereas others did not undergo biopsy of the enlarged lymph node. Meanwhile, one patient was diagnosed with plasma cell granuloma of lung 2 years after the diagnosis of sinonasal IMT, and another patient was diagnosed with sinonasal and orbital IMT 6 years after the diagnosis of middle ear plasma cell granuloma. However, whether it was a multifocal disease or a metastasis was unclear.

Among all the 13 patients, 2 underwent ^18^F-fluorodeoxyglucose positron emission tomography/computed tomography (PET/CT) because of suspicion of a malignant tumor. However, PET/CT could not distinguish IMT from malignant tumor, with the maximal standard uptake value (SUVmax) of the lesion 7.0 and 13.7, respectively. Similarly, five patients underwent radionuclide bone imaging and four of them showed increased radionuclide uptake in the maxillofacial bone.

Regarding the laboratory test results before treatment, erythrocyte sedimentation rate (ESR) of seven patients ranged from 1 to 111 mm/h (71.4% above the normal range), C-reactive protein (CRP) level of 10 patients ranged from 0.51 to 181.26 mg/L (80% above the normal range), and white blood cell count of 12 patients ranged from 3.59 to 17.26×10^9^/L (33.3% with leukocytosis and elevated neutrophil ratio).

### 3.3. Histological Characteristics and Immunohistochemical Staining

**Figure 6:**
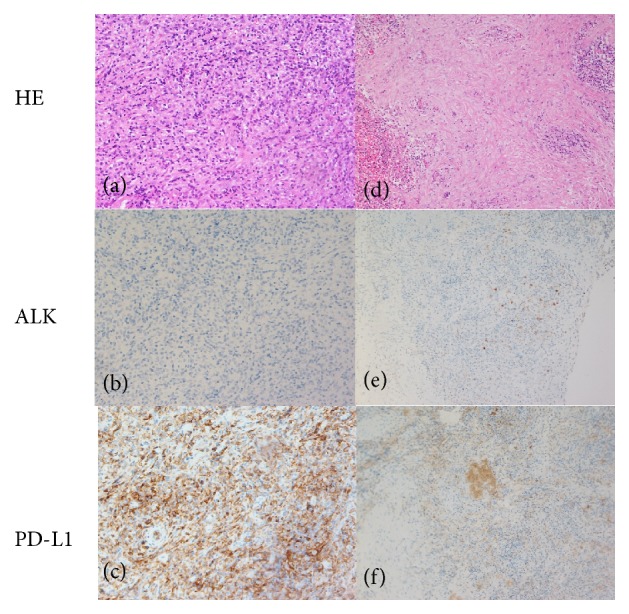
HE and immunohistochemical staining pictures of an ALK negative and PD-L1 positive case (Case 2) were shown in (a), (b), and (c) (original magnification ×200). Another ALK positive and PD-L1 positive case (Case 7) was shown in (d), (e), and (f) (original magnification ×100).

IMT comprises proliferated spindle cells and a large number of inflammatory cells mainly including plasma cells and lymphocytes (shown in Figure 6). Since this was a retrospective study, the available immunohistochemical staining results were not enough. Among the patients, 1/12 (8.3%) was positive for ALK, though weakly, 9/12 (75%) were positive for PD-L1 (shown in Table 2); 8/8 (100%) were positive for CD20; 7/8 (87.5%) were positive for alpha-smooth muscle actin (SMA); 4/4 (100%) were positive for Vimentin. Further, 7/7 (100%) were negative for AE1/AE3, and 8/9 (88.9%) were negative for S-100. The Ki-67 proliferative index ranged from less than 1% to 30% in seven patients. Four patients were immunostained for immunoglobulin IgG4, with two patients weakly positive. Meanwhile, the IgG4/IgG ratio was less than 10% in these four patients.

### 3.4. Treatment and Follow-Up Data

**Figure 7:**
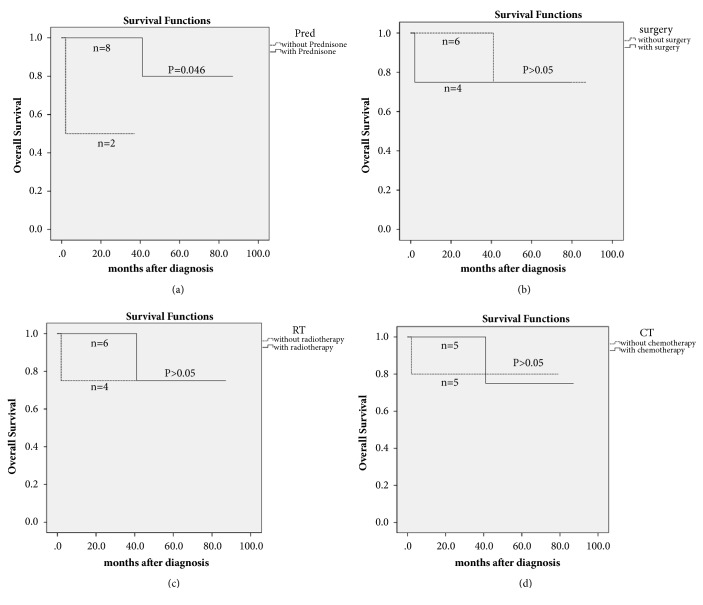
The overall survival (OS) of 10 patients followed up was estimated using the Kaplan–Meier method. Among prednisone (a), surgery (b), radiotherapy (c), and chemotherapy (d), only treatment with prednisone was significantly associated with better OS.

All 13 patients underwent biopsy and were diagnosed with IMT histopathologically by experienced pathologists. The follow-up data was available for 10 patients with the follow-up period ranging from 2 to 87 months (median, 39 months). Among the 10 patients followed up, 2 died of the disease (one was dead of intracranial invasion 2 months after the surgery, and cervical lymph node metastasis was proved; the other one was dead of local progress without metastasis), whereas the other 8 were stable (alive with disease, but there are various degrees of improvement and no disease progression). The treatment and follow-up data are presented in Table 2. Five patients underwent partial resection of tumor followed by different combinations of prednisone, radiotherapy, and chemotherapy, with three patients stable after 12–79 months of follow-up and two patients lost to follow-up. Another five were treated with prednisone combined with chemotherapy and/or radiotherapy. One of them died of the disease 41 months after the diagnosis, and the other four patients were stable after 22–87 months of follow-up. One patient was suspected of a local recurrence of fibrous histiocytoma involving the maxillary sinus and pterygopalatine fossa 1 year after the operation. She underwent expanded maxillectomy and reconstructive surgery, with sinonasal IMT and lymph node metastasis confirmed by postoperative pathology, but she died of intracranial invasion 2 months later. One patient was observed conservatively, but no symptomatic progress was observed 37 months after the diagnosis of parapharyngeal space IMT. Overall, the 5-year survival rate was 72%. The correlation between the treatment and OS was analyzed (Figure 7). The use of prednisone was significantly correlated with better OS (*P* = 0.046). However, treatment with surgery, radiotherapy, and chemotherapy was not significantly associated with OS (*P* > 0.05). Cytoxan (CTX) was used for chemotherapy combined with vincristine in four patients and CTX alone in two patients.

## 4. Discussion

The present study showed no specificity in symptoms of IMT, mainly including local and systematic symptoms. Extrapulmonary IMTs are usually accompanied by constitutional symptoms, where fever, anemia, and weight loss are common in the pediatric cohort as well as in this study [5, 6]. Previous studies showed that IMTs of the nasal cavity, paranasal sinus, and nasopharynx are usually more aggressive compared with those in other anatomical locations [7, 8]. But we are not sure if IMTs in the nasal cavity are more aggressive because of the biology or if the location and local therapy in the head region are more complicated. For most patients in the present study, the tumor invaded only surrounding structures, except two patients having coexisting plasma cell granuloma in other organs (lung and middle ear, respectively). A multifocal disease or a metastasis could be used to explain the coexisting plasma cell granuloma. Radiologically, IMT often presents as an extensive soft-tissue mass with no clear demarcation between the tumor and adjacent normal structures. Bone destruction and enhancement on contrast-enhanced CT or MRI were also seen in the present study and a previous study [9]. Some changes in laboratory test results were also found, suggesting the inflammatory process with elevated ESR, increased CRP, leukocytosis, and elevated neutrophil ratio, which was similar to the findings of a previous study [6]. The inflammation values of one patient who died were slightly increased; however, the other one died and the stable case without any therapy had normal inflammation values. Therefore, it is hard to say whether the systemic inflammation reaction is associated with the prognosis. A previous retrospective study of FDG PET/CT findings in six patients with IMT in other anatomic locations showed that the mean SUVmax was 10.9 ± 5.5 and the high variability might be due to tumor cellularity, biological behavior, and the proportion and activation of inflammatory cells [10]. In the present study, two patients underwent PET/CT scan with SUVmax 7.0 and 13.7, which was consistent with the result of the previous study. Four of five patients showed an increased radioactive concentration of the maxillofacial bone in radionuclide bone imaging, implicating the invasion of bone. Therefore, PET/CT and radionuclide bone imaging might play some role in tumor detection and follow-up, although the results could not distinguish IMT from a malignant tumor. IMT should be differentiated from malignant tumor to make a more appropriate treatment plan. Therefore, a biopsy is crucial when IMT or malignant tumor is suspected. Some patients underwent repeated biopsies before the diagnosis was confirmed in this study.

The main treatment regimens of IMT in the present study included surgery, glucocorticoids, chemotherapy, and radiotherapy, which were used alone or in combination empirically. A previous study suggested that patients with resectable IMT were usually treated surgically [11]. However, the inoperable (given damages to function and appearance after the operation) IMT of the paranasal sinuses and orbit with recurrence responded well to prednisolone and chemotherapy [12]. Another previous review also recommended the use of oral high-dose corticosteroids (prednisone, 60–100 mg per day, which was tapered slowly) as the first-line therapy for plasma cell granuloma of the head and neck [7]. In the present study, one patient underwent radical resection and died 2 months after the surgery because of intracranial invasion. Among others treated more conservatively, only one patient died of the disease 41 months after diagnosis. Therefore, it was speculated that radical operation might stimulate the growth of IMT. Recent studies showed that ALK-positive IMTs were likely to respond to the targeted therapy with ALK inhibitors [13, 14]. However, IMTs in the paranasal sinus were less likely to be detected with ALK rearrangement compared with those in other locations, as shown in a previous study [15]. Only one patient was positive for ALK immunohistologically in the present study, but without identification of gene rearrangement. Other kinase fusions in addition to ALK were identified in IMT, which were targeted with tyrosine kinase inhibitors, through the next-generation sequencing- (NGS-) based genomic assay [16]. Latest studies showed that IMTs expressed constitutive and adaptive PD-L1 frequently, especially in ALK-negative tumors, suggesting new therapeutic targets with anti-PD1/PDL1 therapy, which is popular in many other types of tumors [17, 18]. The positive rate of PD-L1 in our study was comparable to that of previous study, so anti-PD1/PDL1 therapy may be used for IMTs of paranasal sinus and nasopharynx.

Regarding the prognosis of IMT in the nasal cavity and paranasal sinus, the 5-year survival rate of the 10 cases followed up in the present study was 72%. Also, treatment with prednisone was significantly correlated with better OS (*P* = 0.046, Figure 7), whereas other treatments were not. However, the results should be confirmed by conducting more randomized controlled studies with a large number of patients and standard intensity of treatment. The present study did not analyze the correlation between pathological characteristics and OS because the data available was not enough. One clinicopathological study of 25 patients with nasal sinus IMT showed that multiple relapse, necrosis, mitosis, ganglion-like cells, and histological patterns were associated with poor outcomes [19]. The relationship between the clinical outcome of IMT and ALK expression is controversial. ALK expression is associated with better outcomes, as shown in some studies [5, 20]. However, other studies suggest that ALK-positive patients are more likely to suffer from local recurrence but not distant metastasis [21] and IMTs with nuclear membrane or perinuclear ALK and epithelioid morphology are more aggressive [22].

The limitations of this study included lack of detailed histologic features, small sample size, and a retrospective design. Also, the correlation between histologic characteristics and prognosis was not available. Moreover, individual dosage and time of treatment, including medication and radiotherapy, were not exactly the same. For most of our cases, different regimes were conducted simultaneously or successively. Therefore, it was difficult to evaluate the exact effect of different treatments, although treatment with prednisone was found to be significantly associated with better OS. And many patients in a stable state after some therapy had not been reevaluated the resectability.

In conclusion, IMT of the paranasal sinus and nasopharynx is an intermediate soft-tissue tumor that often mimics malignancy. We are not sure if IMTs in the nasal cavity are more aggressive because of the biology or if the location and local therapy in the head region is more complicated. For unresectable IMT, combined therapy with prednisolone, chemotherapy, and radiotherapy is sometimes a better choice. Glucocorticoids are especially recommended as a basic part of the integrated therapy. The standard treatment for nasal IMT needs further research with the advent of novel treatment and more follow-up data.

## Figures and Tables

**Table 1:** Clinical characteristics.

Clinical characteristics	

Gender	
Male	*n* = 6 (46%)
Female	*n* = 7 (54%)
Age	
Range	21-64 years
Mean	40.7 years
Symptoms	
Facial swelling and pain	*n* = 9 (69%)
Trismus	*n* = 6 (46%)
Headache	*n* = 5 (38%)
Toothache	*n* = 5 (38%)
Decreased vision	*n* = 4 (31%)
Diplopia	*n* = 3 (23%)
Protopsis	*n* = 3 (23%)
Fever	*n* = 3 (23%)
Weight loss	*n* = 6 (46%)
Anemia	*n* = 5 (38%)
Common sites	
Maxillary sinus	*n* = 10 (77%)
Orbit	*n* = 9 (69%)
Ethmoid sinus	*n* = 8 (62%)
Nasal cavity	*n* = 5 (38%)
Infratemporal fossa	*n* = 5 (38%)
Pterygopalatine fossa	*n* = 4 (31%)
Parapharyngeal space	*n* = 4 (31%)
Sphenoid sinus	*n* = 4 (31%)
Nasopharynx	*n* = 4 (31%)

**Table 2:** Treatment and follow-up data.

Case number	Main sites	Treatment	ALK	PD-L1	Months after diagnosis	Outcome
1	PS	IR + Pred + RT	-	-	79	Stable
2	PS, NP, orbit	IR +Pred + CT	-	+	/	Lost
3	PS, IF	Pred + CT + RT	-	+	41	DOD
4	PS, orbit	Pred + CT + RT	-	-	87	Stable
5	PS, orbit	IR + Pred + CT	-	+	12	Stable
6	PS, IF	Pred + CT + RT	-	-	50	Stable
7	PS, NP	IR + Pred + RT	+	+	20	Stable
8	PS, orbit	Pred + CT	-	+	62	Stable
9	NC, PS	IR + RT	/	/	/	Lost
10	NP	Pred + RT	-	+	22	Stable
11	PS, orbit, SB	UK	-	+	/	Lost
12	PS, SB	CR	-	+	2	DOD
13	Parapharyngeal space	Observation	-	+	37	Stable

CR, Complete resection; CT, chemotherapy; DOD, died of disease; IF, infratemporal fossa; IR, incomplete resection; NC, nasal cavity; NP, nasopharynx; Pred, prednisone; PS, paranasal sinus; RT, radiotherapy; SB: skull base; Stable, alive with disease, but there are various improvements and no disease progression; UK, unknown.

## Data Availability

The data used to support the findings of this study are available from the corresponding author upon request.
